# Improving cause of death certification in the Philippines: implementation of an electronic verbal autopsy decision support tool (SmartVA auto-analyse) to aid physician diagnoses of out-of-facility deaths

**DOI:** 10.1186/s12889-021-10542-0

**Published:** 2021-03-22

**Authors:** Rohina Joshi, R. H. Hazard, Pasyodun Koralage Buddhika Mahesh, L. Mikkelsen, F. Avelino, Carmina Sarmiento, A. Segarra, T. Timbang, F. Sinson, Patrick Diango, I. Riley, H. Chowdhury, Irma L. Asuncion, G. Khanom, Alan D. Lopez

**Affiliations:** 1 The George Institute for Global Health, University of New South Wales, Sydney, Australia; 2 The George Institute for Global Health, New Delhi, India; 3 School of Population and Global Health, University of Melbourne, Parkville, VIC Australia; 4 Epidemiology Bureau, Department of Health, Manila, Philippines

**Keywords:** SmartVA, Verbal autopsy, SmartVA for physicians, Cause of death, Medical certification of cause of death

## Abstract

**Background:**

The majority of deaths in the Philippines occur out-of-facility and require a medical certificate of cause of death by Municipal Health Officers (MHOs) for burial. MHOs lack a standardised certification process for out-of-facility deaths and when no medical records are available, certify a high proportion of ill-defined causes of death. We aimed to develop and introduce SmartVA Auto-Analyse, a verbal autopsy (VA) based electronic decision support tool in order to assist the MHOs in certifying out-of-facility deaths.

**Method:**

We conducted a stakeholder consultation, process mapping and a pre-test to assess feasibility and acceptability of SmartVA Auto-Analyse. MHOs were first asked to conduct an open-ended interview from the family members of the deceased, and if they were not able to arrive at a diagnosis, continue the interview using the standardised SmartVA questionnaire. Auto-Analyse then presented the MHO with the three most likely causes of death. For the pilot, the intervention was scaled-up to 91 municipalities. We performed a mixed-methods evaluation using the cause of death data and group discussions with the MHOs.

**Results:**

Of the 5649 deaths registered, Auto-Analyse was used to certify 4586 (81%). For the remaining 19%, doctors believed they could assign a cause of death based on the availability of medical records and the VA open narrative. When used, physicians used the Auto-Analyse diagnosis in 85% of cases to certify the cause of death. Only 13% of the deaths under the intervention had an undetermined cause of death. Group discussions identified two themes: Auto-Analyse standardized the certification of home deaths and assisted the MHOs to improve the quality of death certification.

**Conclusion:**

Standardized VA combined with physician diagnosis using the SmartVA Auto-Analyse support tool was readily used by MHOs in the Philippines and can improve the quality of death certification of home deaths.

**Supplementary Information:**

The online version contains supplementary material available at 10.1186/s12889-021-10542-0.

## Article summary

- Our study demonstrates that SmartVA Auto-Analyse can be readily used as a decision support tool for physicians to certify out-of-facility deaths in the absence of good quality medical records.
- The key element for the successful implementation of this study was the leadership and governance of the Philippines Department of Health who were closely involved in the planning and implementation of the intervention.
- Understanding the significance of the intervention, and integrating the intervention within the workflow enabled acceptance by the MHOs and communities.
- The study lacks a direct comparison of SmartVA Auto-Analyse with Philippine Statistical Authority data and it would have been useful to compare SmartVA Auto-Analyse with death certificates prior to cleaning and coding by Philippine Statistical Authority.
- The successful pilot project has led to the Government deciding to expand the use of SmartVA Auto-Analyse to the entire country in a phased fashion.

## Introduction

For registration of deaths in the Philippines Civil Registration and Vital Statistics (CRVS) system, all deaths need a death certificate with an assigned cause of death [1]. Deaths that occur in health facilities are certified by the attending physician. In the case of an out-of-facility death, the current practice is that municipal health officers (MHOs), medical graduates with minimum 3 years of experience, complete the death certificate based on an interview with family members of the deceased. By law, only family members can request for the death certificate. For the certification of out-of-facility deaths, comprising 60% of all deaths [2], no training is provided to the MHOs regarding interviewing the family or certifying the death. Each MHO uses their own method of certification, which results in high inter-physician variation and the frequent use of codes such as senility, cardio-respiratory arrest, and death due to ‘other ill-defined and unspecified causes’, none of which are useful for guiding public policy. Indeed, in 2016, about one in five home deaths were assigned a cause that could not be an underlying cause of death [3].

The Philippine Government recognizes the importance of having a well-functioning CRVS system that can reliably be used to guide policy debates about priorities for health services delivery and provide planners with reliable data on deaths and causes of death. The Philippines is a signatory to the Regional Action Framework (RAF) of United Nationals Economic and Social Commission for Asia and the Pacific’s Decade to improve CRVS and is part of the Data for Health Initiative [4]. The overall aim of the RAF is to strengthen the CRVS system and have universal registration of births and deaths in the region by 2024 [5]. Philippines has set as a national goal to have at least 85% of those deaths that take place without medical attention certified by using an internationally acceptable verbal autopsy (VA) method.

VA is a method used to determine the most likely cause of death using information about the signs and symptoms surrounding the death from family members of the deceased. Verbal autopsy method usually provides the underlying cause of death defined as ‘the disease or injury which initiated the train of morbid events leading directly to death’. The questionnaires are typically administered by community healthcare workers. The symptom and demographic information gathered has traditionally been analyzed by physicians but has increasingly been analyzed by algorithms that improve the consistency and accuracy of diagnosis at no cost [6].

One such method is SmartVA which was originally developed by the Population Health Metrics Research Consortium based on a ‘gold standard’ validation data set of over 12,500 cases from several countries, including the Philippines [7]. The questionnaire was subsequently shortened and comprises four modules, (i) a general module with questions on age, sex and demographic details, (ii) neonate module (0–27 days) (iii) child module (28 days to 11 years) (iv) adolescent and adult module (12 years and above). Each questionnaire includes structured symptomatic questions with multiple-choice answers which originally was followed by an unstructured open-ended narrative section. The structured part comprises a list of check boxes and responses and the unstructured section involves the interviewer asking the respondent to describe in their own words the symptoms that occurred before the death, recording the narrative as it flows [8].

The questionnaire was further adapted by a team at the University of Melbourne in collaboration with a Technical Working Group (consisting of the Department of Health and the Philippine Statistical Authority) for use by physicians. In this paper, we describe the development, testing, evaluation and potential of this adapted VA instrument, ‘SmartVA Auto-Analyse’, for improving the diagnostic accuracy of community deaths in the Philippines and other countries where physician input into the certification of community deaths is required.

## Methods

SmartVA Auto-Analyse was developed and implemented in six steps:

### Stakeholder consultation

A Technical Working Group comprising stakeholders from the Department of Health, Philippines Statistics Authority, Municipal Health Officers, national and international researchers with expertise in verbal autopsy methods, and implementation partners was established to decide upon the study methods. The consultation process involved reviewing different VA tools, understanding technology requirements, training needs and budget required for implementation.

### Workshop with physicians and site visits

Next, sites visits were conducted to better understand the death certification and registration process for community deaths and assess the time required by families and MHOs to process the paper work. A preliminary workshop was also held with MHOs to demonstrate the SmartVA tool and discuss the current challenges faced by the MHOs during the certification process.

### Process mapping

The process of certification and registration of a death was mapped separately for community and hospital deaths. This was undertaken to understand all the steps involved in registering and certifying deaths, identifying responsibilities for government offices and individuals, and the inter-relationships between the various steps and CRVS actors.

### Pre-test

This step was carried out in order to understand potential implementation and technology challenges. Since a physician-based model for the implementation of SmartVA had not been evaluated before, a feasibility study (pre-test) was conducted in 14 municipalities and cities in one of the eight official languages of Philippines (Tagalog). The pre-test was evaluated using quantitative and qualitative methods. Twenty physicians were trained in SmartVA and correct procedures for the medical certification of cause of death. In addition, seven IT personnel and four support staff were trained in the technical aspects of SmartVA such as setting up a tablet and trouble-shooting, if needed. MHOs were provided with an android tablet to record the interviews during a period of 4 months. Five hundred twenty-six cases were collected, almost all adult deaths. The ODK Collect Android application used the shortened SmartVA questionnaire and was analyzed by the Smart VA Auto-Analyze 1.2 software (Tariff 2.0). An evaluation workshop with the MHOs at the end of the pre-test was conducted to assess the cause specific mortality fractions (CSMFs) that were obtained for plausibility, as well as group discussions to understand the barriers experienced in using SmartVA-Analyze to diagnose the cause of death. Results from the evaluation suggested that physicians used the SmartVA diagnosis in 65% of the cases certified (*N* = 342). When the MHOs did not use the SmartVA-Analyze diagnose, it was generally because they either used available medical records or gleaned additional diagnostic information from the interview that enabled them to make a more specific diagnosis, such as changing chronic kidney disease to diabetes mellitus, or assigning cases of ‘other non-communicable diseases’ to specific conditions such as rectal cancer. Overall, the MHOs reported that SmartVA-Analyze was a very useful diagnostic aid but preferred to ask the open-ended questions before the structured ones in the SmartVA instrument, consistent with their clinical training.

### Development of ‘*Auto-Analyse’*

**Fig. 1:**
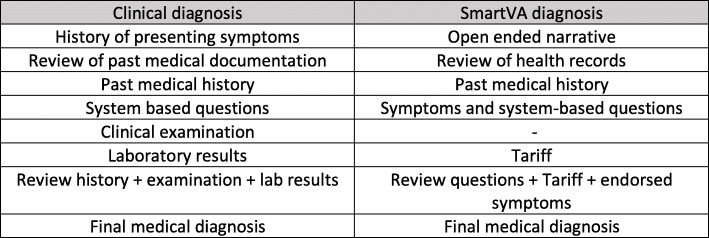
Alignment of *SmartVA for physicians* to the MHO’s clinical thought process

**Fig. 2:**
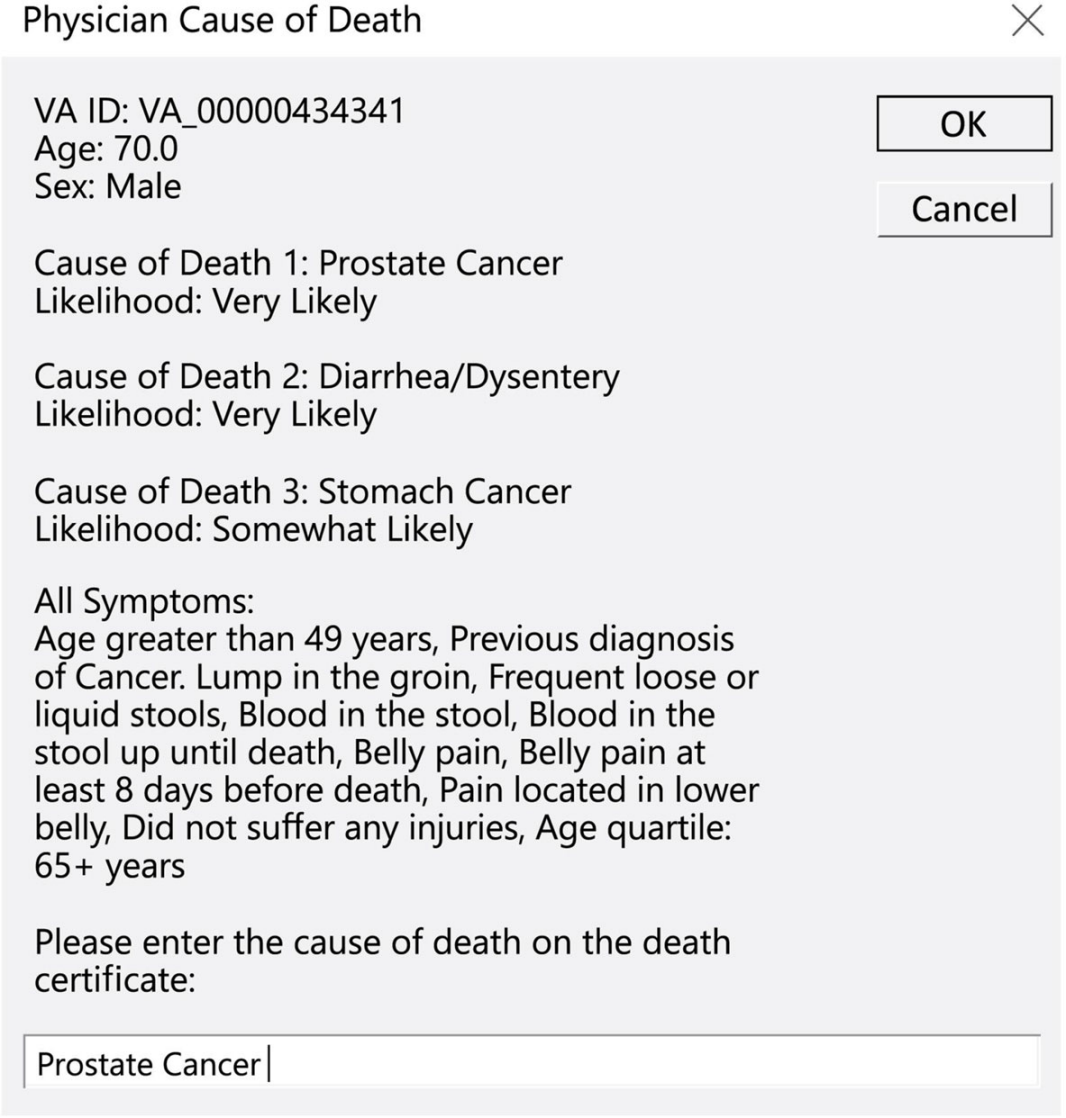
Screenshots of SmartVA Auto-Analyse

**Fig. 3:**
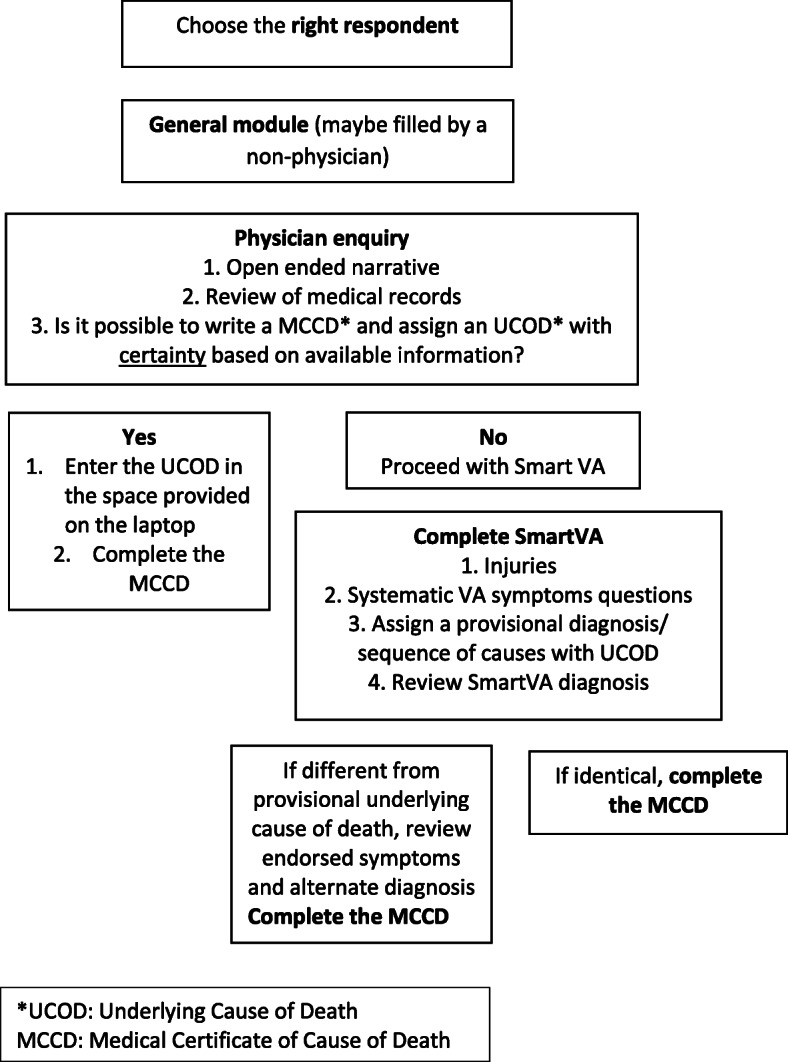
Standard operating procedure for *SmartVA for Physicians*

Another important modification was that the SmartVA-Analyze VA software was changed to ‘SmartVA Auto-Analyse’ for real-time use by physicians and to produce a list of the top three most likely causes of death, as well as the full list of endorsed diagnostic symptoms used for prediction. Taking the results of the pre-test into account, the SmartVA questionnaire was modified to align it with the clinical and logical thought process of physicians to certify deaths (Fig. 1). The key adaptation of ‘SmartVA Auto-Analyse’ was to change the sequence in which the different VA sections were presented to certifying physicians, commencing with the open-ended narrative and past history, followed by the system based structured questions. SmartVA Auto-Analyze 1.2 software was used to analyze the modified questionnaire [9]. Another addition was to provide the MHO with a convenient summary of all the endorsed symptoms (i.e. symptoms that the family reported at interview) as well as the top three most likely causes of death as predicted by SmartVA-Analyze, including the likelihood of dying from each cause (possible, somewhat likely, likely, very likely) as determined from the tariff score produced by the algorithm (Fig. 2). The likelihood was based on the relative ranking of the tariff scores that met the necessary cause-specific and absolute thresholds [9]. If no causes met the threshold, then an undetermined cause was presented to the MHO. The MHO could then agree with one of the SmartVA-Analzye causes, based on his or her assessment of available medical records and the family interview, or else conclude a separate diagnosis when certifying the cause of death. A standard operating procedure was developed to enable the MHOs to decide when to use Auto-Analyse to certify deaths, and the SmartVA manual was adapted accordingly (Fig. 3).

### Pilot with mixed-methods evaluation

The ‘Auto-Analyse’ questionnaire and software output were translated and cognitively tested in a further two languages (Ilonggo and Cebuano) and piloted in 91 municipalities and cities in seven regions. One hundred twenty-six MHOs were trained in the use of Auto-Analyse and medical certification of cause of death in three batches, each of which lasted for 3 days. Furthermore, IT personnel were trained to install and troubleshoot any problem with the software. Manuals and training guides were distributed during the training sessions and newsletters were distributed quarterly to monitor and report on progress.

#### Data management and analysis

**Fig. 4:**
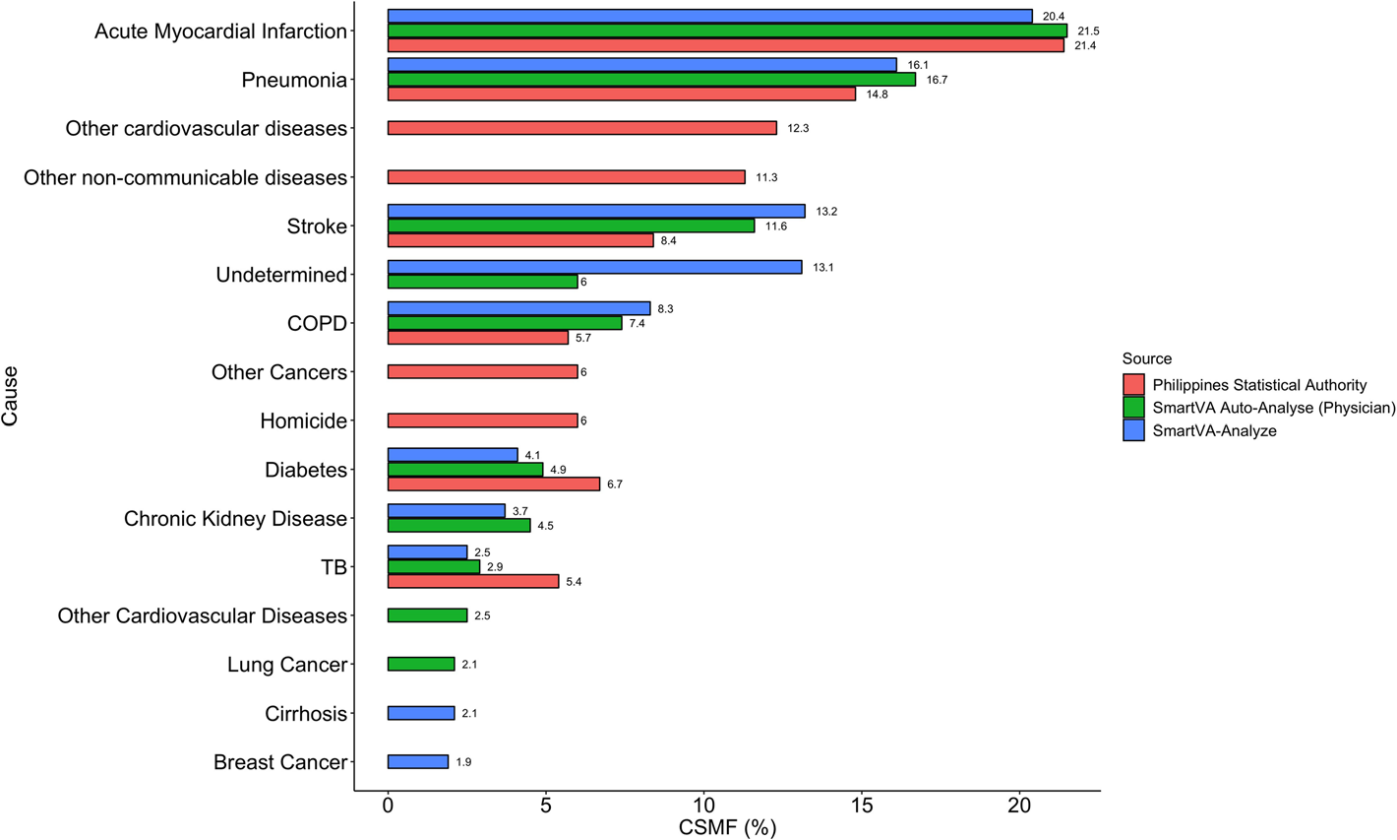
Leading causes of death by SmartVA-Analyze, SmartVA Auto-Analyse, and Philippine Statistical Authority, 2018

Each week MHOs sent an excel spread sheet of de-identified data to the Study Coordinator who compiled the data, including up to three Auto-Analyse assigned causes of death and the physician assigned underlying cause of death. Quantitative analysis was based on the de-identified data set. National mortality data from the Philippines Statistical Authority [10], which collects data for all facility and home deaths, was also tabulated and mapped to corresponding cause categories (Fig. 4) for comparison with the ‘Auto-Analyse’ and physician-determined cause of death.

Qualitative analysis was based on the focus group discussions conducted with 40 MHOs and 20 non-physicians at the end of the pilot study. Workshop participants were divided into three groups with a facilitator to guide the discussion on the following topics: 1) had Auto-Analyse helped in certifying the cause of death (COD) and what could be enhanced in future implementation? 2) Technology – what works and what can be improved?; and 3) what is missing or should be strengthened in the current training provided? (see Additional file 1).

#### Ethics

Since this was a Government initiative which was part of routine practice, formal ethics approval was not required. All the physicians invited to take part in the pilot gave their verbal consent to participate. No identifiable data were collected for the purposes of this study.

#### Patient and public involvement statement

There was no patient or public involvement in the development of the intervention. Stakeholders included representatives from the Department of Health, Philippine Statistical Authority and Municipal Health Officers from the pilot study located in Manila.

## Results

During the period January to June 2018, 5649 community deaths were reported, diagnosed and registered in the pilot areas (55% male, 45% female). Auto-Analyse was used to certify the cause of 81% (4586 out of 5649) of these community deaths during the pilot phase. For the remaining 19%, doctors were confident that they could assign a cause of death based on the availability of medical records and asking additional questions to substantiate the diagnosis. Medical records were available for 1914 (33.4%) of all deaths. Interestingly, even when medical records were available, physicians often reported to have used SmartVA to aid in the diagnosis of cause of death. The explanation for why SmartVA was used for 69% of all deaths with medical records could of course be that the information available was not relevant or sufficient for a diagnosis to be made. We were unable to access and review the quality of medical records used by the MHOs. Due to issues in data entry (such as blanks and incorrectly entered data), the final set of deaths used for the evaluation was 4376. Almost all the respondents (99%) were family members of the deceased.

More than 70% of the 126 MHOs reported using the Auto-Analyse tool regularly to assist them in certifying the cause of death. The majority of interviews were conducted by physicians or a combination of MHOs and their assistant non-physicians. In some cases, MHOs delegated the task of interviewing the relatives to a nurse or mid-wife and reviewed the endorsed symptoms to certify the death. Most of the remaining 30% or so of MHOs were either supervisors or regional office staff who do not certify deaths routinely, or had experienced technological issues, such as use of a Macintosh Computer, or the tablet and laptop were not compatible.

The top 10 causes of death among the community deaths investigated using SmartVA-Analyze are presented in Fig. 4. Acute myocardial infarction (AMI) (21%) was the leading cause of death followed by pneumonia (17%) and stroke (12%). 13% of deaths were undetermined. The majority of undetermined deaths occurred in the older age group (65% were over the age of 60 years) where it often is challenging to identify an underlying cause of death due to the number of co-morbidities commonly present at or around the time of death [11]. When physicians used Auto-Analyse as a decision support tool to assign an underlying cause of death, AMI was the leading cause in (20%), followed by pneumonia (16%), and stroke (13%). Only 13% of all deaths could not be assigned a specific cause of death when using Auto-Analyse (Fig. 4). While Auto-Analyse could reliably distinguish certain cancer types (breast, leukemia/lymphoma, lung, esophageal, stomach, colorectal, prostate, cervical) and combine the other cancers into a common category (other cancers), the physicians were able to further classify cancer into 22 sites by asking specific questions of the respondent.

The leading causes of death from both SmartVA-Analyse and SmartVA Auto-Analyse were comparable to those published by the Philippine Statistical Authority with AMI (21%), Pneumonia (15%), and Stroke (8%) being the leading specific causes of death (Fig. 4). Despite these similarities, it should be noted that the two data sets are not strictly comparable, as the Philippine Statistical Authority data includes all home deaths and not only those for which there were no medical records. It seems reasonable to expect that many of those for which the MHOs had no supportive evidence before SmartVA would have ended up with an unreliable cause.

Of the 781 deaths in which physicians assigned a different cause than SmartVA-Analyze, physicians most frequently disagreed with the SmartVA-Analyze diagnosis of stroke (19%), acute myocardial infarction (19%), and pneumonia (13%). There was no discernible age pattern of disagreement. Physicians most commonly reclassified SmartVA -diagnosed stroke deaths into AMI (31%), Other Cardiovascular diseases (19%), and Diabetes (19%);, AMI deaths into Diabetes (25%), Other Cardiovascular (22%), and Stroke (22%);, and pneumonia deaths into Chronic Obstructive Pulmonary Disease (21%), Tuberculosis (14%), and Diabetes (11%). The least amount of reclassification was for drowning and suicide deaths, where both physicians and SmartVA-Analyze identified the same 14 and 10 deaths, respectively.

The MHOs could easily integrate Auto-Analyse into existing routine processes without significantly adding to their workload. The average time taken to complete the Auto-Analyse interview and diagnosis could not be measured precisely as interviews were often interrupted and some doctors used IT staff to transfer the questionnaire to the computer and run the Auto-Analyse software. However, from the focus group discussions, most reported a length of interview ranging from 10 to 30 min depending on the cases and the knowledge of the interviewed family member.

### MHO perspectives regarding the use of SmartVA

The focus group discussions confirmed that the MHOs overwhelmingly found SmartVA Auto-Analyse to be a useful tool for assisting them with medical certification of deaths. In particular, they found the structured interview to be a helpful and convenient guide in asking questions. The summary list of the endorsed symptoms and the list of the top three most likely causes of death was reported to be helpful in deciding the most probable cause of death. The MHOs reported that the interview questions triggered their thought process to ask extra questions that often allowed them to provide a diagnosis that was more specific than the one offered by SmartVA-Analyze. For example, it was mentioned that it became possible to further disaggregate the SmartVA-Analyze cause ‘other cancers’ into more specific sites such as ‘nasopharyngeal cancer’. Many MHOs felt that even if medical records were available, it was useful to use Auto-Analyse to confirm, and compare diagnoses, thereby increasing the amount of critical diagnostic acumen brought to bear on the certification process.

## Discussion

Our study demonstrates that SmartVA Auto-Analyse can be readily used as a decision support tool for physicians to certify deaths that occur in out-of-facility in the absence of medical records, or where medical records are poor. The tool was acceptable by physicians and community members and could be readily integrated into diagnostic practices already used for completing the death certificate. Based on other information available to them, MHOs agreed with the Auto-Analyse diagnosis in 81% of cases, and in cases where MHOs disagreed with the Auto-Analyse diagnosis, the standardized instrument provided the physicians with a framework for arriving at a diagnosis. This pilot project demonstrates that Auto-Analyse can strengthen the health information system for community deaths in the Philippines by providing some evidence for diagnosis and eventually reducing the fraction of ill-defined causes of death and thus, increasing their information content for policy.

The key element for the successful implementation of this study was the leadership and governance of the Philippines Department of Health who were supportive of the intervention and closely involved in the implementation from the beginning of the pre-test and pilot. While the time taken to use SmartVA often was more than their previous practice (approximately 10–15 min), they appreciated that the additional evidence and standardization provided by the instrument was helpful and would result in improvement in quality of the death certificate and data for health policy.

Systematic application of a validated VA tool [12] helped decrease inter-physician variability and reduced the fraction of causes coded to “Other ill-defined and unspecified causes of mortality” (R99), Cardiac arrest (I46) and ‘Senility’ (R54) to only 6% compared to 13% for use of SmartVA-Analyze and approximately 18% before the intervention. Giving the physicians three options to choose from (i.e. the three most probable causes of death diagnosed by the algorithm) and a summary of the symptoms reported by the family helped them make a logical clinical decision based on the available evidence and gave them confidence to assign an underlying cause of death.

The greatest disagreement between the physicians and SmartVA-Analyze was for stroke and AMI deaths. Cardiovascular causes of death often have multiple comorbidities and the physicians may have felt constrained by the single cause of death that SmartVA-Analyze outputs. Indeed, the majority of other disagreements for these causes, such as mistaking stroke for AMI, fall within the same broad cause group. Some of the disagreements were due to more specific causes than VA can produce, such as more specific cancer diagnoses.

Physicians showed high agreement with many external causes that SmartVA-Analyze predicted. Auto-Analyse helped provide a structured interview to determine the cause of these deaths and proved to be useful also for external causes. While it may be more difficult to glean diagnostic information from older deaths which have multiple morbidities, Auto-Analyse is useful as a decision support tool regardless of the age of death.

The Philippine Statistical Authority cause of death data was used as a comparison with the physician and SmartVA-Analyze assigned cause of death. All data sources identified the same specific leading causes of death, but the Philippine Statistical Authority information identified more deaths due to residual causes (e.g. “other cardiovascular”, “other non-communicable”). Some of the deaths that the Philippine Statistical Authority assigned to residual causes may have been undetermined by the physicians and SmartVA-Analyze. Additionally, the Philippine Statistical Authority data are not directly comparable to community deaths predicted by Auto-Analyse because they are an amalgamation across the country that have been cleaned and coded. Moreover, the community death comparator from the Philippine Statistical Authority amalgamates those that had medical records and those without, i.e. those that are easy to certify and those that are not, while the VA data are essentially consists of the latter only. Nonetheless, the results here suggest that the physician assigned cause of death with the help of Auto-Analyse are comparable to existing data sources. We acknowledge this limitation, as a robust comparison of the two methods would be to review the death certificates prior to cleaning and coding.

Verbal autopsy methods have evolved overtime and are now being implemented in countries in routine practice, beyond research settings [6, 13]. The Data for Health Initiative is one such large collaboration which aims to strengthen the data collection, analysis and use of cause of death data in countries. One of the main objectives of the initiative is to understand system related issues, provide technical resources, and promote the integration of VA data into CRVS systems, enhancing the use of data for maximum impact in policymaking and priority setting [4]. Most low- and middle-income countries (LMICs) train community health workers to interview families and either train physicians to analyse the cause of death [14] or use automated methods to analyse the cause of death at the population level [15–17]. While analysis by computer algorithms has proven to be more effective that physician VA alone, the combination of the computer VA and physician diagnosis leverages the advantage of the consistency of the computer with the local knowledge and the ability for the physician to provide a more specific diagnosis. The Philippines is the first country in the world that uses an electronic decision support system specifically designed to help doctors arrive at a cause of death for community deaths. In fact, the government recently issued an Administrative Order making VA part of the certification for out-of-facility deaths. Other countries (Colombia, China, Peru) are now following Philippine’s lead and using a similar model for certification of deaths [18]. As countries aim to improve their data for community deaths, this intervention is a realistic and achievable option [19].

The main lessons learnt from this novel intervention was the importance of supportive leadership and good governance, both from the implementers and technical partners. Local ownership with collaborative partnerships that include health, statistics, registration and policy makers is the key to successful implementation [20]. Understanding the significance of the intervention and integrating the intervention within the workflow enabled acceptance by the MHOs and communities. The successful pilot project has led to the Government deciding to expand the use of SmartVA Auto-Analyse to the entire country. They plan to do so in a phased fashion and monitor the progress overtime.

## Conclusions

With the availability of validated verbal autopsy tools and technological developments, verbal autopsy is increasingly being considered as a valuable and feasible approach to strengthen national CRVS systems in LMICs. The Philippines is a good example of a country which has demonstrated how its current human resources and infrastructure can be adapted to strengthen its health information system using SmartVA, thereby improving the quality of CoD data to guide health policy formulation and evaluation.

## Supplementary Information

**Additional file 1.**

## Data Availability

The data that support the findings of this study are available from the civil registration and vital statistics system of The Philippines, but restrictions apply to the availability of these data, which were used under license for the current study, and so are not publicly available. For further details, please contact the corresponding author.

## Abbreviations

AMI: Acute myocardial infarction
CoD: Cause of death
CSMFs: cause specific mortality fractions
CRVS: Civil Registration and Vital Statistics
LMICs: Low- and Middle-income countries
MHO: Municipal Health Officers
RAF: Regional action framework
SmartVA: Smart verbal autopsy
VA: Verbal autopsy
