# Supplementary material for: Improving cause of death certification in the Philippines: implementation of an electronic verbal autopsy decision support tool (SmartVA auto-analyse) to aid physician diagnoses of out-of-facility deaths
Source: BMC Public Health. 2021 Mar 22;21:563. doi: 10.1186/s12889-021-10542-0 (PMC7986549; doi:10.1186/s12889-021-10542-0)
Supplement: Supplementary file 1 — Additional file 1. [file 12889_2021_10542_MOESM1_ESM.docx]

**Topics for the group discussions with Municipal Health Officers**

- Topic 1: SmartVA implementation challenges and facilitators (gains & pains) – before and after SmartVA
  - Thinking about implementation (not only technology) What worked for you? What didn’t?
  - Did SmartVA help you in certification – explain?
  - What could have been done better?
  - Give 3-5 practical recommendations about SmartVA. How can DoH and D4H support you better.
- Topic 2: Technology - what works and what can be improved (discussed in light of Roger's presentation) – before and after SmartVA
- Topic 3: National roll out of SmartVA – what is missing in the current training? how and where should it be rolled out? Are we training the right people? Is the effort worth the gain? What was done before?
  - Thinking about the training received, give 5 practical recommendations about training. Think about your own clinic, who does the interview, who should be trained?
